# Acute and Transgenerational Effects of Non-Steroidal Anti-Inflammatory Drugs on *Daphnia magna*

**DOI:** 10.3390/toxics11040320

**Published:** 2023-03-29

**Authors:** Anna Michalaki, Konstantinos Grintzalis

**Affiliations:** School of Biotechnology, Dublin City University, D09 N920 Dublin, Ireland

**Keywords:** *Daphnia magna*, non-steroidal anti-inflammatory drugs, NSAIDs, indomethacin, ibuprofen, toxicity, immobilization, enzyme activity, feeding, transgenerational

## Abstract

Pharmaceuticals pose a great threat to organisms inhabiting the aquatic environment. Non-steroidal anti-inflammatory drugs (NSAIDs) are major pharmaceutical pollutants with a significant presence in freshwater ecosystems. In this study, the impact of indomethacin and ibuprofen, two of the most commonly prescribed NSAIDs, was assessed on *Daphnia magna*. Toxicity was assessed as the immobilization of animals and used to determine non-lethal exposure concentrations. Feeding was assessed as a phenotypic endpoint and key enzymes were used as molecular endpoints of physiology. Feeding was decreased in mixture exposures for five-day-old daphnids and neonates. Furthermore, animals were exposed to NSAIDs and their mixture in chronic and transgenerational scenarios revealing changes in key enzyme activities. Alkaline and acid phosphatases, lipase, peptidase, β-galactosidase, and glutathione-S-transferase were shown to have significant changes in the first generation at the first and third week of exposure, and these were enhanced in the second generation. On the other hand, the third recovery generation did not exhibit these changes, and animals were able to recover from the induced changes and revert back to the control levels. Overall, our study points towards transgenerational exposures as more impactful laboratory studies to understand pharmaceutical stressors with a combination of molecular and phenotypic markers of physiology.

## 1. Introduction

Pharmaceutical compounds pose a greater risk to the aquatic environment and human health due to bioaccumulation and human activities [1,2,3]. Because of the global increase in human population and, as a result, the increase in elderly people, the use of pharmaceuticals is becoming highly significant. Pharmaceuticals end up in the environment as a result of their widespread consumption, where they are extremely difficult to biodegrade [3,4]. Pharmaceutical compounds have been observed in potable and groundwater, surface water, sewage treatment plants, Waste Water Treatment Plants (WWTPs), soils, and sediments [5,6]. Pharmaceuticals are primarily derived from industrial or domestic wastewater, improper disposal of expired drugs, and products used in farming and aquaculture [3]. Furthermore, pharmaceutical metabolites and their subsequent transformation products may enter the environment via the WWTP process [2,3,4]. Besides that, biotic or abiotic processes such as hydrolysis or photolysis can degrade pharmaceuticals further [4,7]. These procedures may result in the production of additional pharmaceutical products that are potentially more toxic than the parent compound [4,8]. As a result, it is critical to develop new, more efficient methods for removing pharmaceutical compounds from the environment [2].

Non-steroidal anti-inflammatory drugs (NSAIDs) are a pharmaceutical class that is commonly found in aquatic ecosystems worldwide, accounting for more than 15% of all pharmaceuticals found in the environment [9,10]. As an outcome, NSAIDs can be detected in concentrations greater than 1 μg/L in wastewater treatment plant (WWTP) influx and outflow and in lower concentrations (ng/L) in surface waters [9]. Even though the concentrations of NSAIDs in freshwater ecosystems are low, their high biological activity may cause significant harm to non-target organisms [9,10]. The constant inflow of NSAIDs from WWTPs poses a serious threat to the aquatic environment and its organisms, which are constantly exposed to these emerging contaminants [10]. NSAIDs can be categorized into two groups based on their COX selectivity: nonselective NSAIDs, which block both COX isoforms (indomethacin and ibuprofen) and COX-2-selective inhibitors [11].

Indomethacin is one of the most potent nonselective NSAIDs available, and it was among the first NSAID medications used to treat migraines and headaches [11]. It is an indole-derivative and one of the most commonly detected NSAIDs due to its widespread use [12]. Additionally, indomethacin belongs to the class of intestinal toxicants that can cause gastrointestinal lesions through direct mucosal irritation and inhibition of prostaglandin synthesis [13]. Despite its therapeutic properties, indomethacin is known for its adverse effects in a percentage range of 30–60% of patients receiving it. These adverse effects can be classified as cardiovascular, gastrointestinal, nervous system, hematologic, ocular and otic, renal and electrolyte, dermatologic and sensitivity reaction, and hepatic [11]. Indomethacin can enter the ecosystem through both direct and indirect routes by being improperly disposed of in the toilet or as an excretion product. Furthermore, traces of indomethacin have been found in wastewater samples from Peterborough, Canada, as well as raw water samples from Bosnia and Herzegovina, Croatia, Serbia, and Romania [3,14]. It has been reported that indomethacin is one of the NSAIDs with the lowest detection levels (<2 μg/L) in Romanian WWTPs influents [3]. This observation is in line with a previous publication, which states that indomethacin poses a less serious risk to the environment due to its low concentrations [15].

Ibuprofen is the third most popular of the NSAIDs used globally and has been included in the WHO (World Health Organization) Essential Drug list 2021 [16,17]. Ibuprofen inhibits cyclooxygenase-1 (COX-1) and cyclooxygenase-2 (COX-2) and has a significant analgesic and antipyretic role [18,19]. Ibuprofen is a derivative of propanoic acid and can be detected in several freshwater ecosystems [17,20,21]. Despite the fact that ibuprofen has significant analgesic and antipyretic properties, excessive or inappropriate use may have negative effects on the gastrointestinal tract, the kidney, and the coagulation system [19]. Ibuprofen can enter freshwater ecosystems indirectly or directly [20]. Due to its inability to be fully metabolized by humans, it enters the ecosystem as an excretion product. The direct way for ibuprofen to end up in the environment is by being improperly disposed of in the toilet [20]. Ibuprofen has been detected at concentrations of 45 μg/L, 1.38 μg/L, and 5.78 μg/L in wastewater in Canada, South Africa, and Belgium, respectively. However, even concentrations of 1.673 mg/L and 1.417 mg/L have been detected in wastewater in Pakistan and in surface waters in China [17,22].

To improve our understanding of water pollution, the use of key species as bioindicators are currently highlighted. The combination of phenotypic and sensitive molecular endpoints is a game changer in ecotoxicology and risk assessment. Such approaches fall under the category of New Approach Methodologies (NAMs), which can supplement current analytical tools with sophisticated molecular endpoints [23]. Daphnids are important freshwater ecosystem components that are commonly used in freshwater ecology and ecotoxicology due to their geographical distribution, central role in freshwater food webs, adaptation to a variety of habitats, and, last but not least, sensitivity to anthropogenic chemicals. Daphnids are simple to culture in the laboratory and serve as a useful model organism for molecular ecotoxicology and mechanistic insight into novel pollutants [24,25,26].

This study aimed to investigate the effects of indomethacin, ibuprofen, and their combined mixture on *D. magna*, specifically through chronic and transgenerational exposure at a non-lethal concentration that is not environmentally relevant, using feeding assay as a phenotypic endpoint and molecular markers of physiology. It should be noted that most studies usually focus on phenotypic endpoints such as mortality in daphnids and usually in acute or chronic exposures but only in one generation. This is the first study to look at the effects of indomethacin, ibuprofen, and their combined mixture on daphnids using molecular markers rather than toxicity or phenotypic markers alone and in-depth in more generations. Moving toward transgenerational studies provide more insight into the relation to imprinted stress and its mechanisms. In this study, the effects of two NSAIDs, indomethacin and ibuprofen, were assessed on *D. magna.* Furthermore, in the actual environment, chemicals are not encountered alone; therefore, for a realistic scenario, their 1:1 mixture was assessed in non-lethal concentrations. The toxicity of these pharmaceuticals and their mixture on daphnids was evaluated using acute toxicity curves. These toxicity curves were constructed based on the mortality of daphnids, which was assessed as immobilization [27]. Following this, their impact on a phenotypic endpoint, feeding rate, was evaluated with a novel approach based on the ingestion of fluorescent microparticles. The impact of indomethacin, ibuprofen, and their mixture on the physiology of daphnids was evaluated using biochemical markers in chronic exposures. The main goal of this study was to assess, even at higher than environmental concentrations, if daphnids are affected and to what extent and if they can actually recover from the stress of exposure when they are subsequently cultured in clean media.

## 2. Materials and Methods

### 2.1. Reagents

All chemicals used in this study were of the highest purity >99.9% and quality. Indomethacin, ibuprofen, KCl, Na_2_SeO_3_, latex beads, carboxylate-modified polystyrene, fluorescent red, bovine serum albumin, brilliant blue G, *p*-nitrophenyl butyrate, 2-nitrophenyl-B-D-galactopyranoside, 1-chloro-2,4-dinitrobenzene, L-glutathione reduced, sodium phosphate dibasic were purchased from Sigma-Aldrich. CaCl_2_·2H_2_O, MgSO_4_·7H_2_O, NaHCO_3_, HCl, *p*-nitrophenyl phosphate, boric acid, ammonium acetate, NaOH, methanol, and DMSO were purchased from Fisher Scientific.

### 2.2. Culturing of Daphnids and Acute Toxicity

Daphnids were cultured under a 16 h:8 h of light:dark photoperiod at 20 °C in OECD media (final concentrations 0.29 g CaCl_2_·2H_2_O/L, 0.123 g MgSO_4_·7H_2_O/L, 0.065 g NaHCO_3_/L, 0.0058 g KCl/L, 2 μg Na_2_SeO_3_/L, pH 7.7) [28]. Indomethacin and ibuprofen were dissolved in DMSO, which had a final concentration of 0.005% *v*/*v* in the exposure, which is within the concentration range used in previous studies [27,29,30]. Neonates (<24 h) were collected from the third brood of their mothers and used for experiments. To assess toxicity, 15 neonates were exposed to each NSAID separately, and their 1:1 mixture in a final volume of 50 mL OECD media with four replicates per concentration tested. Based on similar studies [21,28,30,31,32,33,34], all assays were conducted with four (toxicity curves and biochemical assays) or five (feeding assay) replicates per condition tested. For toxicity, the OECD guidelines state that at least 40 animals (preferably in four groups of 10 animals should be used at each test concentration); however, in our study, we exceeded that number to 60 animals. Toxicity curves were plotted for 24 h exposures, and EC values were calculated using the four-parameter logistic (4PL) model, following the equation Span = Top − Bottom and Y = Bottom + (Top − Bottom)/(1 + 10^((LogIC50 − X) × HillSlope)), using the GraphPad software. The parameters top and bottom were commonly fixed to 100 and 0, accordingly. Mortality in daphnids was assessed as their immobilization [27].

### 2.3. Feeding Assay and Imaging

**Figure 1:**
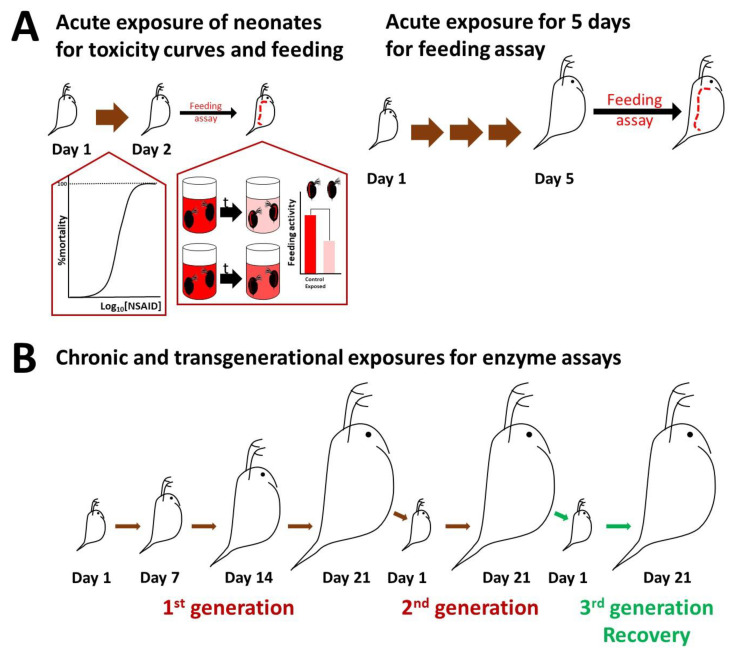
Experimental design. (**A**) Acute exposures in neonates and five-day-old daphnids to assess mortality and feeding. (**B**) Chronic and transgenerational exposures followed by a recovery generation for the quantification of enzyme activities. Figure created with BioRender.com. Brown arrows indicate exposure to NSAIDs and their combined mixture, while green arrows show recovery exposure in the absence of NSAIDs.

The feeding rate was assessed in neonates, which were exposed to NSAIDs at a non-lethal concentration of 1 mg/L for 24 h or cultured until five days old (Figure 1A). To assess the feeding rate of neonates, D1 daphnids were exposed for 24 h to indomethacin, ibuprofen, and their mixture at 1 mg/L. After 24 h, twenty daphnids were transferred in a 12-well plate with 6 mL OECD containing the carboxylate-modified polystyrene, fluorescent red microparticles (2.0 μm mean particle size) at a concentration of 13 mg/L. The animals were exposed to the microplastic for up to 90 min, and media was collected every 15 min to estimate the removed microparticles by fluorescence at Ex 560 Em 590 nm using a TECAN plate reader. The concentration of microparticles in the media was optimized to ensure an excess of microparticles for the accurate quantification of ingestion. Furthermore, we have extensively tested these particles, which show no toxicity to the daphnids for the short exposure periods used. We performed control experiments with neonates, as they would be more sensitive, for even up to 10 h to microplastics at even higher concentrations (up to 52 mg/L), and all animals from all experiments following the feeding assay were alive and healthy. Fluorescence was expressed to the amount of ingested microparticles using a standard curve. To confirm the ingestion of microparticles in the gut of daphnids, animals were imaged with a stereoscope and fluorescence microscopy using the TRITC filter for carboxyl functionalized particles. For D5 daphnids, neonates were exposed for 5 days to indomethacin, ibuprofen, and their mixture at 1 mg/L in 600 mL OECD. The animals were fed daily with 2 mL of fresh algae (*Chlamydomonas reinhardtii*) with a concentration of 6 million cells/mL. On the 5th day of exposure, five daphnids were brought to a 12-well plate with 6 mL OECD containing the fluorescent red microparticles at a concentration of 13 mg/L. Daphnids were allowed to ingest the microparticles for 60 min, and then the same procedure as described above was followed (Figure 1A).

### 2.4. Biochemical Assays

Chronic exposure was performed at 1 mg/L of each NSAID and their 1:1 mixture separately, which was selected as a non-lethal concentration. For enzyme activities in chronic and transgenerational exposures, neonates were cultured for two consecutive generations and following a 21-day recovery period (Figure 1B). Twenty neonates (<24 h) were exposed for 7, 14, and 21 days to indomethacin, ibuprofen, and their mixture at 1 mg/L in 600 mL OECD media. Media and NSAIDs were renewed twice a week. Although the concentration of NSAIDs was not measured throughout the exposure, their renewal twice a week was sufficient to exert their action. Furthermore, indomethacin has been shown to be stable for up to 12 days at room temperature [35,36]. There are many reports stating that ibuprofen is stable for 15 days at room temperature [37,38]. Ibuprofen can maintain >90% average strength for 5 months, according to [39], and half-life (t_1/2_) for more than a year (t_1/2_ > 1 year) at 25 °C [40]. Daphnids were fed daily with 2 mL fresh algae (*C. reinhardtii*) with a concentration of 6 million cells/mL, and a seaweed extract (*Ascophylum nodosum*) was supplemented only on the days of media change. Neonates from the D21 daphnids (of the first generation) were exposed to the same conditions for 21 days as the second-generation exposure. For recovery, neonates from the second generation were cultured only in OECD media in the absence of NSAIDs for 21 days as a recovery generation.

Five 7, 14, and 21-day-old animals were used per biological replicate and homogenized in 0.5 mL ddH_2_O using a pestle homogenizer. The homogenate was cleared by centrifugation (12,000× *g* for 5 min at 5 °C), and the clear supernatant was collected and used to assess the enzyme activity as described elsewhere [28,41]. 200 μL appropriately diluted sample in buffer was assessed for the activity of phosphatases (in 100 mM acetic acid pH 4.5 for acid phosphatase or 100 mM boric acid pH 9.8 for alkaline phosphatase) using 50 μL of the substrate *p*-nitrophenyl phosphate (8 mM) and monitoring the production of *p*-nitrophenol at 405 nm after its alkalinization (with 50 μL 4M NaOH). In addition, the activities of β-galactosidase and lipase were measured with the same experimental conditions by the generation of nitrophenol from the catalysis of *o*-nitrophenyl-β-galactoside or *p*-nitrophenyl butyrate, respectively, but in phosphate buffer pH 7.2. Glutathione-S-transferase (GST) activity was assessed by the reaction of reduced glutathione with 1-chloro-2,4-dinitrobenzene. 200 μL appropriately diluted sample in phosphate buffer pH 7.2 was mixed with 50 μL 2 mM CDNB, and 6 mM reduced glutathione, and the formation of the complex was measured continuously at 340 nm and converted to units of activity with the extinction coefficient [42,43]. Protein was quantified by an ultrasensitive Bradford protocol to normalize the results [44].

### 2.5. Statistical Analysis

The biochemical data were presented as mean ± standard deviation and analyzed with the GraphPad Prism software. Statistically significant differences were compared by Student’s *t*-test over unexposed control with a *p* value of 0.05 for chemical exposures.

## 3. Results

### 3.1. Acute Toxicity of NSAIDs and Their Mixture

**Figure 2:**
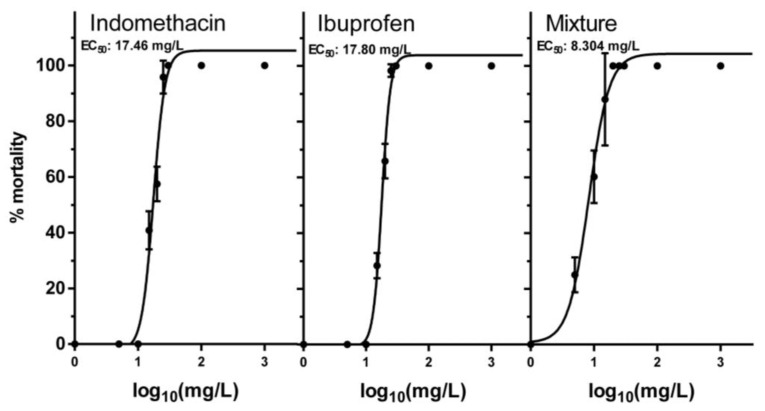
Acute toxicity curves in neonates for indomethacin, ibuprofen, and their mixture. Data represent average ± standard deviation (N = 4 replicates).

Acute exposures of neonates to NSAIDs, indomethacin, ibuprofen, and their mixture were assessed via toxicity curves (Figure 2), and the effective concentration (EC) values were calculated (Table 1). The EC_50_ values for indomethacin and ibuprofen were very close to each other, 17.46 mg/L and 17.80 mg/L, respectively. A 1:1 mixture for both NSAIDs was also tested for mortality and showed a lower EC_50_ of 8.304 mg/L, indicating a potential synergy of the two NSAIDs. A non-lethal concentration of 1 mg/L was chosen for both NSAIDs and their mixture for chronic exposures.

### 3.2. Feeding Assay and Imaging

**Figure 3:**
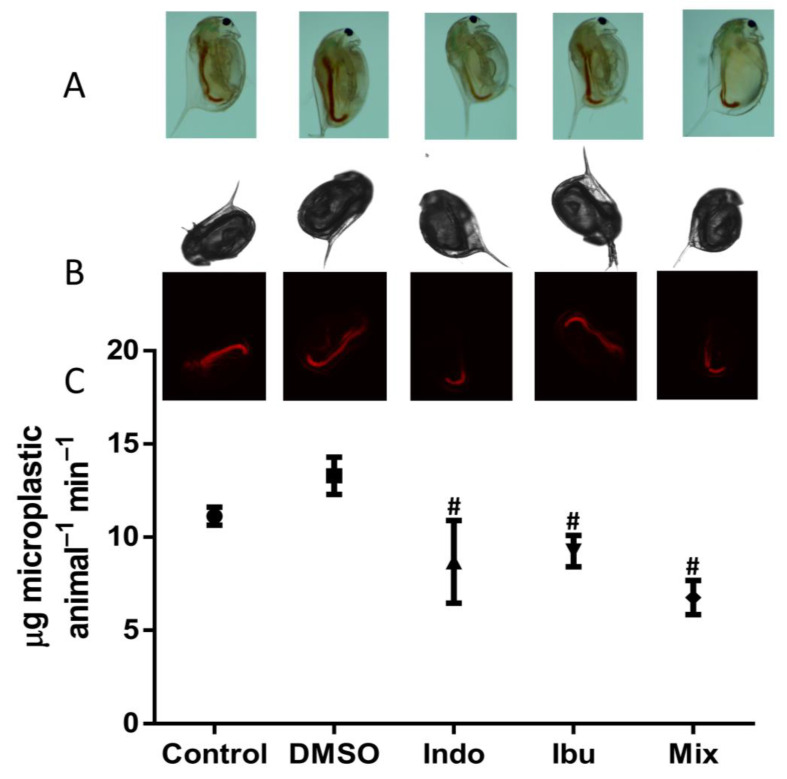
The impact of NSAIDs on feeding rate to neonates. The ingested microparticles were visualized by optical stereoscopy (**Panel A**), bright field, and fluorescence microscopy (**Panel B**). Feeding rate was quantified by the ingestion of microparticles based on their fluorescence in the incubation media (**Panel C**). Data represent average ± standard deviation (N = 5 replicates). # Statistically significant by Student’s *t*-test denotes significant difference in comparison to the DMSO carrier solvent.

**Figure 4:**
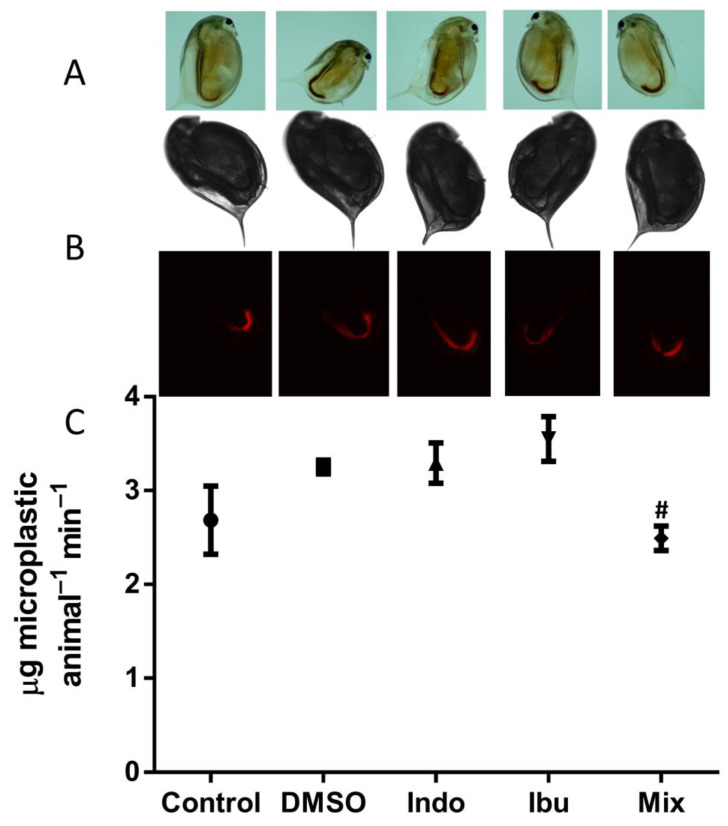
The impact of NSAIDs on feeding rate to D5 daphnids. The ingested microparticles were visualized by optical stereoscopy (**Panel A**), bright field, and fluorescence microscopy (**Panel B**). Feeding rate was quantified by the ingestion of microparticles based on their fluorescence in the incubation media (**Panel C**). Data represent average ± standard deviation (N = 5 replicates). # Statistically significant by Student’s *t*-test denotes significant difference in comparison to the DMSO carrier solvent.

Feeding is a phenotypic endpoint used to evaluate the physiology of daphnids as a non-invasive test [34]. We used particles with a mean particle size of 2.0 μm because *D. magna* feeds non-selectively on a wide range of particles with sizes ranging from 1 to 50 μm [45]. These particles were selected as they would allow tracking with fluorescence microscopy and were not toxic to daphnids. The feeding rate was determined by the ingestion of fluorescent microparticles in neonates and five-day-old daphnids (Figure 3 and Figure 4). The ingestion of microplastics was confirmed using stereoscopy, bright field, and fluorescent microscopy. Exposure of neonates to indomethacin, ibuprofen, and their composite mixture reduced the feeding rate by 35%, 30%, and 49%, respectively, when compared to DMSO (Figure 3). However, when five-days-old animals were exposed, a different pattern was observed, and only their mixture decreased the feeding rate by 23% (Figure 4). This can be potentially explained as the animals are older and more resistant to the stress of NSAIDs when compared to neonates.

### 3.3. Enzymatic Activity following Chronic and Transgenerational Exposure of Daphnids to NSAIDs

Chronic exposure to 1 mg/L of indomethacin, ibuprofen, and their mixture resulted in significant changes in the physiology of daphnids during their growth (Table 2). Following the first 7 days of exposure, indomethacin significantly reduced the activities of alkaline phosphatase (ALP), β-galactosidase (β-gal), and lipase (LIP), while ibuprofen and their mixture decreased the activities of only the latter two. After 14 days of exposure, the only enzyme which showed a change in activity was β-gal by 28% in the NSAID mixture. Exposure for one more week resulted in a decrease in the activity of acid phosphatase (ACP) for indomethacin and the NSAID mixture. In contrast, indomethacin increased the activities of β-gal and peptidase (PEP), whereas ibuprofen and their mixture increased the activity of glutathione-S-transferase (GST). The first generation of exposures was continued for additional 21 days in daphnids for the second generation. Indomethacin inhibited the activities of LIP, PEP, and GST, whilst ibuprofen inhibited the activities of ALP, β-gal, LIP, and PEP. Ibuprofen also increased the activity of GST. Apart from GST, the NSAID mixture had an impact on all enzymes. Except for ACP, the activities of ALP, β-gal, LIP, and PEP decreased. Finally, daphnids following their second generation of exposure were transferred for 21 days in OECD media as a third generation of a 21-day recovery. PEP and GST were the only enzymes with increased activity during the recovery period. Indomethacin and ibuprofen increased the activity of GST by 19% and 6%, respectively, and their mixture increased the activity of PEP by 16%, thus showing that the recovery period in clean media allowed the animals to return to a control condition for the majority of the enzymes. The general conclusion is that initial exposure had an impact on markers of physiology, while during the period the animals prepare for fertility (14 days), this was alleviated and followed more changes in the first generation over 21 days, which were even stronger in the second generation. Finally, for the recovery, it was observed a return to the control condition.

## 4. Discussion

The acute and chronic effects of indomethacin, ibuprofen, and their mixture were assessed using a combination of physiological indicators, such as mortality and feeding, as well as biochemical markers. As biochemical markers, we assessed the activity of phosphatases, β-galactosidase, lipase, peptidase, and glutathione-S-transferase. NSAIDs, such as ibuprofen, diclofenac, and naproxen, can form reactive oxygen species (ROS). The disruption of the balance between ROS and the antioxidant systems in the organism is referred to as oxidative stress. When oxidative stress occurs, it causes lipid and protein peroxidation, damage to DNA structure, as well as inhibition of digestive enzymes (trypsin, β-galactosidase) [27,46]. According to these reports, ibuprofen increases lipid peroxidation, protein carbonyl content (protein oxidation), and enzymes of antioxidant defense SOD and CAT. As a result, lipid peroxidation affects differently different types of enzymes, such as phosphatases and lipases. Additionally, the oxidation of proteins affects the activity of proteins such as peptidases, β-galactosidase, lipases, and phosphatases [47]. Glutathione-S-transferase belongs to a group of enzymes involved in the detoxification processes [48]. An increase in this enzyme might indicate that the daphnids use these detoxification processes in order to adapt and survive the stress that the chemicals cause [49]. Most NSAIDs are toxic to organisms due to their bioaccumulation in the ecosystems. There are a few studies that have been published about the impact of NSAIDs, such as indomethacin and ibuprofen, on *D. magna*, but the majority of them rely on mortality, growth, and reproduction rate to determine toxicity [21,29,31,50,51]. Extending to other aquatic species, it has been noted that indomethacin and ibuprofen can harm other marine and freshwater organisms.

Indomethacin has been shown to significantly increase the biomass of *Chironomous riparius*, while it had no effect on the survival rate or biomass of *Physella acuta* [52]. After 24 h of exposure, lethal concentrations for *Thamnocephalus platyurus*, *Oryzias latipes*, and *D. magna* were 16.14 mg/L, 81.92 mg/L, and 22.38 mg/L, respectively [3,5]. There are not sufficient data on indomethacin toxicity in daphnids, although the findings of the two aforementioned studies [3,5] are very close to our lethal concentration. The toxicity of indomethacin in other species, such as male zebrafish (*Danio rerio*) for 96 h, was in a similar order of magnitude with an EC_50_ of 76.30 mg/L. However, indomethacin has been shown to cause significant changes in the transcriptome of zebrafish in marker genes such as superoxide dismutase 1, glutathione peroxidase 1, interleukin-1, tumor necrosis factor-alpha, and others [13].

Ibuprofen has been described in the literature as a substance with high mobility in aquatic ecosystems. Despite the fact that up to 90% of ibuprofen can be removed from the environment efficiently, it is present in high concentrations in raw sewage [20,21]. It has been reported that chronic exposure of neonates *D. magna* to concentrations of 20, 40, and 80 mg/L for 14 days affected somatic growth, reproduction, and survival [21]. Somatic growth, in particular, increased with increasing concentrations. There was a significant delay in fecundity and a total decrease in reproduction in daphnids exposed to 40 mg/L and 80 mg/L, respectively. Finally, survival of daphnids exposed to 20 mg/L and 40 mg/L was unaffected, yet it was reduced by 19% in daphnids exposed to 80 mg/L [21]. These findings are consistent with previous reports, which tested the same concentrations of ibuprofen to *D. magna* for a 10-day period [50]. Ibuprofen had a similar dose-dependent effect on reproduction and survival [50]. Exposure of adult daphnids (14 days old) for 8 days to 20, 40, and 80 mg/L ibuprofen showed a decrease in fecundity at concentrations greater than 20 mg/L. However, the brood release was not postponed. The broods produced after exposure to ibuprofen at 80 mg/L consisted of almost dead and/or undeveloped embryos [31]. Grzesiuk exposed *D. magna* to ibuprofen over six generations and found that exposed daphnids had higher growth rates than unexposed daphnids in the 6th generation, which agrees with the findings of Heckmann and Hayashi. In addition, 20%, 70%, and 20% of the animals in the first generation of exposed daphnids produced abnormal offspring. These percentages increased significantly to 80%, 90%, and 50% of individuals in the fifth generation, respectively. They also discovered undeveloped embryos, neonates missing an eye, and daphnids with malformed antennules and carapace. All these abnormalities are considered lethal, reinforcing the claim that even low concentrations of ibuprofen in the environment might have significant impacts on *D. magna* [20]. The toxicity of ibuprofen has been studied in other crustaceans, including *T. platyurus. T. platyurus* was exposed for 24 h to a range of concentrations ranging from 0.1 to 66.7 mg/L. The lethal concentration after 24 h of exposure was calculated to be 19.59 mg/L. This EC closely matches our findings, while the EC for ibuprofen, according to our study, is 17.80 mg/L [5]. It has also been reported in the literature that chronic exposure to ibuprofen at concentrations of 0.0001, 0.05, 1, 8, and 25 mg/L affects some phenotypic markers of zebrafish (*Danio rerio*), such as spontaneous movement, free swimming distance, duration and speed under dark condition, as well as enzymatic markers, such as the activity of GST [53,54]. Specifically, 28-day exposure of zebrafish to 25 mg/L ibuprofen caused an increase in the activity of GST [53]. Furthermore, ibuprofen caused toxic effects on the mussel *Mytilus galloprovincialis* [55,56], the crayfish *Procambarus clarkii* [57], the marine clam *Ruditapes philippinarum* [58], the clam *Corbicula fluminea* [59] and to the mussel *Dreissena polymorpha* [60].

Given the scarcity of research on the effects of indomethacin and ibuprofen on freshwater organisms, particularly *D. magna*, our study aimed to highlight their significance while also introducing additional markers of physiology other than mortality, growth, and reproduction rate as common endpoints. We recently demonstrated that markers related to physiology and enzymatic activity, in addition to metabolic perturbations, are useful endpoints for pollution assessment [28,30]. It is worth noting that this is the first reference of indomethacin, ibuprofen, and daphnids using additional molecular markers rather than using only toxicity results.

According to our findings, both indomethacin and ibuprofen, as well as their mixture, were toxic to daphnids. Even though 1 mg/L is not an environmentally relevant concentration (ng/L to μg/L), there are many reports that tested concentrations of NSAIDs significantly higher than 1 mg/L [21,27,29,30,31,50,51,61]. In our setup, the concentration of NSAIDs was not measured during chronic exposures. Monitoring the concentration could provide valuable insight regarding their stability in water. Nevertheless, there are reports indicating that indomethacin and ibuprofen can be stable for 12 and 15 days at room temperature, respectively [35,36,37,38]. A novel approach to assessing the feeding rate was used as an alternative approach when compared to other feeding assays [62,63]. To date, the feeding assays that are being used rely on algae counts, although these approaches employ large volumes of media or longer feeding periods (4 to 24 h) [62,63,64,65]. Microparticles, on the other hand, are uniformly defined by the manufacturer, and thus they offer flexibility in fluorescence, whereas algae are only fluorescent due to chlorophyll. Moreover, because microparticles differ from chlorophyll or other potentially fluorescent compounds, they can be easily identified and visualized inside the animals using microscopy. Therefore, this approach allows the measurement of the microparticles in the media missing and in the animal present. Another benefit of this test is that it is immediate and fast, allowing the process of a large number of samples in a high throughput manner. For these reasons, as well as the high reproducibility, our previously published method [34] has been optimized to a microparticle approach. In terms of the toxicity of microparticles, while particles, in general, are toxic to daphnids, this is accurate for long periods of exposure and at higher concentrations [66]. The toxicity of the microplastics used in our study was assessed using a toxicity curve (part of another research paper), which revealed that these particles are toxic only at extremely high concentrations and over a longer exposure period. Polystyrene microplastics were toxic to *D. magna* at concentrations higher than 30 mg/L for neonates and 100 mg/L for adults, as well as during longer exposure periods (>96 h) [45].

Indomethacin, ibuprofen, and their mixture appear to affect feeding rates differently in neonates and D5 daphnids. When compared to DMSO, the two NSAIDs and their mixture reduced the feeding rate in neonates by 35%, 30%, and 49%, respectively. Diclofenac is also known to reduce the feeding rate in neonates of *D. magna* [67,68]. The assessment of the feeding rate in D5 daphnids, on the other hand, revealed that only the mixture affected, in particular, decreased the feeding rate by 23%. Exposure of D5 daphnids to ketoprofen did not affect the feeding [67]. In chronic exposures of D7 daphnids, indomethacin, ibuprofen, and their combination reduced enzyme activities, although activities of all enzymes except β-gal were not affected after 14 days of exposure. One plausible explanation is that D14 daphnids are older and thus more resilient to indomethacin and ibuprofen. Another possibility is that D14 daphnids are entering their reproductive stage and preparing to release their first large brood [69]. After 21 days of exposure, indomethacin, ibuprofen, and their mixture impacted a number of key enzymes. It has been reported that ibuprofen caused an upregulation in the activity of triacylglycerol lipase [31]. On the contrary, no increase in the activity of lipase was observed in our study. Other NSAIDs, such as diclofenac or acetylsalicylic acid, did not affect the daphnids in the same way. For example, chronic exposure of daphnids to diclofenac reduced the activity of ALP and ACP, while increasing the activity of PEP [30]. Acute exposure of daphnids to acetylsalicylic acid only decreased the activity of PEP [28]. In our study, we observed a reduction of ALP caused by indomethacin on D7 daphnids and by ibuprofen and their mixture on D21 daphnids of the second generation. The activity of ACP was decreased by indomethacin and their mixture on D21 daphnids. The activity of PEP was increased by indomethacin on D21 daphnids and decreased by indomethacin and their mixture on D21 daphnids of the second generation. Finally, the activity of PEP increased by their mixture on D21 daphnids of recovery generation. Ibuprofen and their mixture increased the activity of GST by 25% on D21 daphnids. These results are in line with the previous publication [70]. However, it has been observed that exposure of *M. galloprovincialis* to ibuprofen for 14 days reduced the activity of GST [71]. Additionally, another NSAID, ketoprofen, increased the activity of GST in *D. magna* when exposed to intermediate concentrations (1.2 and 6 μg/L) [67]. Daphnids may have used this detoxification enzyme as a way to survive the stress that the chemicals caused [49]. However, a more significant effect was observed in the second generation of D21 daphnids. This effect was mostly reversed by the third recovery generation in clean media, with the exception of GST activity. In a number of transgenerational studies in daphnids, stress has been shown to be more prominent with the increase of the generation applied, and this transgenerational inheritance has been shown to be environmentally induced by epigenetic marks [72].

## 5. Conclusions

In conclusion, the acute, chronic, and transgenerational effects of indomethacin, ibuprofen, and their mixture were investigated on *D. magna* using a combination of physiological indicators and biochemical markers. The results revealed that these NSAIDs and their combined mixture impacted the feeding rate, as well as the activity of enzymes associated with digestion and detoxification processes in two generations of *D. magna*. Despite the fact that the chosen concentration of 1 mg/L was higher than environmentally relevant levels, the 14-day exposure of daphnids to these NSAIDs and their mixture did not impact the activity of several enzymes of metabolism in these animals. A plausible explanation for this outcome is that D14 daphnids are transitioning into their reproductive phase and preparing to release their first large brood [69]. As a result, the effect of these chemicals cannot be detected on D14 daphnids even though the pollutants are still present. Furthermore, the third generation of daphnids was able to recover from the stress induced by these pharmaceuticals and their mixture in the previous generations. Therefore, even if the previous generations were exposed to the pollutants, the impact is not reflected in the recovery generation, despite the fact that the concentration used was greater than environmentally relevant. Consequently, our findings indicate that some pollutants may become undetectable even by bioindicator species once they enter the aquatic environment. Moreover, our research aimed to highlight the importance of species with plasticity and responsive mechanisms, with the aim to develop new metrics for pollution assessment using daphnids as an equivalent to the canary in the coal mine [73]. This study is a preliminary pilot work for a coming work on environmentally relevant levels but in the depth of exposures to more generations, thereby showing that we need to emphasize long-term exposures in this species.

## Figures and Tables

**Table 1:** EC values (in mg/L) from toxicity curves.

Chemical	EC_50_	(Min–Max)	Hill Slope	EC_1_	EC_5_	EC_10_	EC_20_
Indomethacin	17.46	16.38–18.61	4.731	6.61	9.37	10.97	13.02
Ibuprofen	17.80	17.30–18.31	6.341	8.62	11.19	12.59	14.3
Mixture 1:1	8.304	7.48–9.21	2.696	1.51	2.78	3.67	4.97

**Table 2:** Biochemical markers of daphnid physiology following chronic and transgenerational exposure to NSAIDs. Data represent mean ± standard deviation (N = 4 replicates) of enzyme activity. Enzyme activity was expressed as units/mg protein for phosphatases, β-gal, LIP, and PEP, and as munits/mg protein for GST. Symbols (#) and (*) indicate statistically significant differences by Student’s *t*-test compared with DMSO and control, respectively.

Age (Days)		Control	DMSO	Indomethacin	Ibuprofen	Mixture
7	ALP	5.45 ± 0.34	7.16 ± 0.4 *	6.22 ± 0.21 #* (−13%)	6.55 ± 0.26 *	7.25 ± 0.34 *
	ACP	5.61 ± 0.44	7.74 ± 1.15	6.82 ± 0.51 *	8.14 ± 0.33 *	9.07 ± 0.72 *
	β-gal	1.66 ± 0.18	1.51 ± 0.04	0.91 ± 0.14 #* (−40%)	1.26 ± 0.06 #* (−17%)	1.41 ± 0.03 # (−7%)
	LIP	131.01 ± 19.67	137.72 ± 2.71	72.94 ± 11.09 #* (−47%)	123.76 ± 2.26 # (−10%)	93.14 ± 6.58 # (−32%)
	PEP	24.57 ± 1.04	23.62 ± 2.53	27.26 ± 5.01	29.54 ± 7.34	21.73 ± 4.54
	GST	91.2 ± 3	97.6 ± 12.3	107.9 ± 11.6	95.6 ± 10	76.7 ± 12.7
14	ALP	2.61 ± 0.02	3.24 ± 0.35	3.05 ± 0.29	2.63 ± 0.14	3.13 ± 0.16 *
	ACP	4.57 ± 0.28	5.35 ± 0.91	6.02 ± 0.71	4.55 ± 0.2	4.39 ± 0.19
	β-gal	2.08 ± 0.08	2.46 ± 0.19	2.23 ± 0.39	2.08 ± 0.75	1.77 ± 0.16 # (−28%)
	LIP	159.59 ± 16.43	141.47 ± 8.09	193.84 ± 25.81	148.12 ± 37.62	140.13 ± 10.69
	PEP	1.99 ± 0.1	2.32 ± 0.29	1.94 ± 0.06	1.93 ± 0.27	1.9 ± 0.12
	GST	132.3 ± 2.7	162.1 ± 12.4 *	144.7 ± 5.4 *	151.7 ± 11.7	151.3 ± 19.2
21	ALP	5.32 ± 0.37	4.86 ± 0.2	5.05 ± 0.17	4.89 ± 0.37	4.69 ± 0.18 *
	ACP	2.96 ± 0.07	3.17 ± 0.09 *	2.84 ± 0.13 # (−10%)	3.09 ± 0.04	2.87 ± 0.11 # (−9%)
	β-gal	6.04 ± 0.31	5.36 ± 0.24 *	5.93 ± 0.19 # (+11%)	5.13 ± 0.25 *	5.05 ± 0.29 *
	LIP	95.93 ± 5.85	81.04 ± 7.29 *	90.86 ± 0.42	78.43 ± 4.27 *	77.29 ± 7.29 *
	PEP	11.41 ± 0.6	9.35 ± 0.43 *	10.82 ± 0.6 # (+16%)	10.52 ± 1.39	9.75 ± 0.59 *
	GST	44.2 ± 3.4	54.2 ± 3.7 *	52.3 ± 1.5 *	67.6 ± 4.4 #* (+25%)	67.7 ± 5.8 #* (+25%)
21 2nd generation	ALP	3.3 ± 0.26	3.64 ± 0.19	3.8 ± 0.18 *	3.27 ± 0.15 # (−10%)	3.09 ± 0.16 # (−15%)
	ACP	3.09 ± 0.09	2.75 ± 0.16 *	2.67 ± 0.15 *	2.78 ± 0.21	3.29 ± 0.19 # (+20%)
	β-gal	4.48 ± 0.24	4.76 ± 0.2	4.7 ± 0.23	3.93 ± 0.22 #* (−17%)	3.8 ± 0.09 #* (−20%)
	LIP	70.46 ± 3.87	66.3 ± 5.65	56.41 ± 3.09 #* (−15%)	47.53 ± 1.66 #* (−28%)	56.53 ± 4.31 #* (−15%)
	PEP	7.43 ± 0.6	7.89 ± 0.56	6.81 ± 0.26 # (−14%)	7.15 ± 0.13	6.44 ± 0.33 # (−18%)
	GST	98.4 ± 4.2	104.3 ± 0.6	94.6 ± 2.9 # (−9%)	123.5 ± 2.1 #* (+18%)	111 ± 5.8 *
21 3rd generation recovery	ALP	4.83 ± 0.36	4.2 ± 0.11 *	4.16 ± 0.24 *	4.55 ± 0.44	4.32 ± 0.28
	ACP	3.42 ± 0.28	2.83 ± 0.08 *	3.21 ± 0.28	3.24 ± 0.47	3.06 ± 0.14
	β-gal	6 ± 0.54	4.75 ± 0.17 *	5.03 ± 0.44 *	4.54 ± 0.24 *	4.97 ± 0.36 *
	LIP	55.14 ± 1.79	53.39 ± 2.08	51.19 ± 2.04 *	53.32 ± 6.51	53.56 ± 3.73
	PEP	6.21 ± 0.45	4.47 ± 0.27 *	5.33 ± 0.49	4.61 ± 0.35 *	5.18 ± 0.41 #* (+16%)
	GST	124.4 ± 11.8	130.3 ± 3.5	154.7 ± 4.2 #* (+19%)	138.3 ± 3.5 # (+6%)	141.8 ± 9.3

## Data Availability

Not applicable.
